# Neuroimmune crosstalk in osteoarthritis pain: from synovial inflammation to pain sensitization

**DOI:** 10.3389/fimmu.2026.1924378

**Published:** 2026-09-02

**Authors:** Zhongyan Mao, Kang Wang, Yanxuan Wu, Gongchang Yu, Bin Shi

**Affiliations:** 1 Shandong Key Laboratory of Biomechanics, Neck-Shoulder and Lumbocrural Pain Hospital of Shandong First Medical University, Jinan, Shandong, China; 2 Department of Respiratory and Critical Care Medicine, The First Affiliated Hospital of Army Medical University, Chongqing, China

**Keywords:** central sensitization, neuroimmune crosstalk, nociceptor, osteoarthritis, pain, peripheral sensitization, synovitis

## Abstract

Pain in osteoarthritis (OA) is not explained adequately by cartilage loss or radiographic severity. Synovial inflammation, tissue damage, and neural plasticity interact across the joint, dorsal root ganglia, spinal cord, and brain to shape the intensity and persistence of symptoms. This mini review examines the synovium as an immune niche in OA pain and follows the signals that connect activated macrophages, fibroblast-like synoviocytes, mast cells, T cells, and damaged chondrocytes with nociceptor sensitization. Cytokines, chemokines, prostaglandins, nerve growth factor, complement fragments, and damage-associated molecular patterns can lower nociceptor thresholds and alter TRP, Piezo, and voltage-gated sodium channel activity. Sensory neurons, in turn, release neuropeptides that affect vascular permeability and immune-cell behavior, creating a bidirectional amplification circuit. Persistent peripheral input may recruit dorsal root ganglion macrophages and activate spinal microglia and astrocytes, although direct evidence for specific central immune mechanisms in human OA remains limited. Therapeutic translation therefore requires more than generalized anti-inflammatory treatment. Mechanism-informed stratification integrating synovitis imaging, soluble biomarkers, quantitative sensory testing, and clinical pain features may help distinguish inflammatory, structural, and nociplastic contributions and guide rational combinations of local immune modulation and neural targeting.

## 1 Introduction

Osteoarthritis (OA) is a whole-joint disease involving articular cartilage, synovium, subchondral bone, ligaments, periarticular tissues, and the nervous system, rather than a disorder caused solely by cartilage wear (1, 2). Pain is its principal clinical burden and the main reason patients seek care, yet pain severity correlates only moderately with radiographic damage. This discordance reflects both the absence of sensory innervation in cartilage and the combined influence of inflammation, mechanical stress, and neural plasticity in innervated joint tissues.

The synovium is a particularly important interface because even low-grade synovitis places immune cells, stromal cells, soluble mediators, blood vessels, and sensory nerve endings in close proximity. At the population level, synovitis is associated with structural progression and pain, although neither relationship is uniform across patients (3). Intra-articular cytokines, chemokines, prostaglandins, growth factors, and matrix-derived danger signals can activate or sensitize nociceptors. Repeated peripheral input can then alter signaling in the dorsal root ganglia (DRG) and spinal pain circuits, while activated sensory neurons feed back onto vascular and immune functions (4, 5).

This framework does not imply that all OA pain is inflammatory. Bone marrow lesions, abnormal biomechanics, muscle weakness, sleep disturbance, psychosocial factors, and established nociplastic mechanisms may dominate in some patients. Neuroimmune communication instead provides a testable link between fluctuating joint inflammation and pain that exceeds visible structural damage (6). This review therefore follows the pathway from the synovial immune niche to nociceptor sensitization, peripheral and central amplification, and mechanism-informed therapeutic stratification.

## 2 Synovial inflammation as the immune niche of OA pain

OA synovitis is generally less intense than rheumatoid synovitis, but it remains biologically active and heterogeneous. Imaging and histology demonstrate lining expansion, vascular change, macrophage accumulation, fibroblast activation, and variable mast-cell and lymphocyte infiltration. Ultrasound- or contrast-enhanced MRI-detected synovitis shows a moderate association with knee pain, supporting an amplifying rather than deterministic role (7). Spatial heterogeneity is equally important: painful and less painful regions within the same knee can contain different fibroblast subsets and inflammatory programs (8).

Macrophages integrate cartilage fragments, calcium-containing crystals, lipids, and cytokines through pattern-recognition and scavenger-receptor pathways. Their outputs include TNF, IL-1β, IL-6, CCL2, prostaglandins, and growth factors that act on fibroblasts, chondrocytes, vessels, and sensory neurons (9). Human tissue data associate impaired synovial innate-immune function with worse pain, arguing against a simple pro-inflammatory versus anti-inflammatory binary (10). Experimental suppression of STAT6-dependent macrophage activation reduces OA pain behavior, but the corresponding pain-driving state has not been validated prospectively in human OA (11). Mast cells may amplify vascular permeability and nociception through histamine, tryptase, cytokines, and neurotrophic signals (12). Human studies associate synovial T-helper-cell infiltration with OA-related pain and disability, but the evidence remains observational rather than causal (13).

Fibroblast-like synoviocytes (FLS) are active organizers of this niche. Activated FLS produce IL-6, chemokines, prostaglandins, matrix-remodeling enzymes, and nerve growth factor (NGF), while also regulating leukocyte retention and fibrosis. Single-cell analysis of painful temporomandibular joint OA supports coordinated shifts across fibroblast, macrophage, endothelial, and other immune cell states during inflammatory pain (14). Aging, obesity, and cellular senescence may further bias the niche toward persistent sterile inflammation (15).

These observations also define the limits of current evidence. Cell states vary with anatomical location, lipid exposure, tissue damage, and disease stage, and most human molecular studies rely on end-stage arthroplasty specimens. Synovitis can fluctuate and does not always change in parallel with pain within an individual (16). Although synovial perfusion, volume, and effusion-synovitis are associated with symptoms in knee and hip OA, they represent only part of the pain phenotype (17–19). The synovium is therefore best viewed as a variable immune amplifier whose impact depends on when and where it is sampled and on the responsiveness of the nociceptive system.

## 3 Immune mediators linking synovitis to nociceptor activation

### 3.1 Cytokine, lipid, and chemokine signaling

Inflammatory mediators increase nociceptor gain through both direct and indirect routes. TNF and IL-1β engage kinase pathways that increase neuronal excitability and stimulate secondary mediator production by FLS and macrophages. IL-6-family signaling acts on sensory neurons as well as stromal and immune cells, whereas prostaglandin E2 (PGE2) lowers activation thresholds through EP receptors. The CCL2-CCR2 axis illustrates this dual action particularly well: CCL2 can excite CCR2-expressing joint afferents and recruit monocytes, and CCR2 blockade reduces established hyperalgesia in experimental OA (20, 21). Related CCL2/CCR2 signaling also shapes macrophage accumulation in mouse DRG (22). These data support a feed-forward chemokine circuit, but clinical efficacy of CCR2 blockade in OA pain remains unproven.

### 3.2 Structural danger signals, crystals, and complement

Structural damage can generate inflammatory signals without direct cartilage innervation. A 32-amino-acid aggrecan fragment activates sensory neurons through Toll-like receptor 2 (TLR2), induces neuronal CCL2, and produces knee hyperalgesia in mice (23). Other matrix fragments and high-mobility group box 1 (HMGB1) can activate pattern-recognition pathways in synovial cells or neurons. Calcium-containing crystals are a distinct and clinically relevant class of joint danger signal: basic calcium phosphate, including hydroxyapatite, and calcium pyrophosphate crystals are common in OA tissues and can activate innate-sensing and inflammasome pathways, thereby increasing IL-1β/IL-18 and secondary stromal mediators (3). Crystal burden is heterogeneous, however, and its presence does not establish that crystal-driven inflammation is the dominant source of pain in an individual patient. Complement fragments provide another link between tissue injury, innate activation, and sensitization (24).

### 3.3 Convergence on nociceptor effector channels

These upstream signals converge on neuronal effector channels. Within the transient receptor potential (TRP) family, TRPV1 and TRPA1 have related but non-interchangeable roles. TRPV1 integrates heat, acidity, NGF, and inflammatory kinase signaling; sensitization shifts its activation range and promotes firing and neuropeptide release. TRPA1 and TRPV1 can form functional complexes that regulate inflammatory sensitization in small-diameter sensory neurons (25). Channel effects are also cell-context dependent: neuronal TRPV1 promotes nociception, whereas experimental activation of macrophage TRPV1 can restrain M1-like polarization (26, 27). Piezo2 is a major mechanotransducer in TrkA-positive nociceptors, and its conditional deletion reduces mechanical sensitization in experimental OA (28). Voltage-gated sodium channels then support action-potential initiation and propagation; Nav1.7 is especially notable because it functions in both sensory neurons and chondrocytes, so analgesic and structural effects must be distinguished during drug development (29).

### 3.4 NGF-dependent remodeling and neuronal feedback

NGF links inflammation to both acute sensitization and longer-term neural remodeling. NGF produced by FLS, macrophages, chondrocytes, and other joint cells binds TrkA, enhances ion-channel sensitivity, changes nociceptor transcription, and promotes nerve sprouting. Neuropilin-1 functions as an NGF co-receptor in human and mouse nociceptors, suggesting a possible route to attenuate NGF signaling without complete ligand neutralization (30). Inflamed or fibrotic synovium may further support aberrant sensory innervation; across rat OA models, altered synovial innervation tracks with fibrosis and hyperalgesia (31).

**Figure 1:**
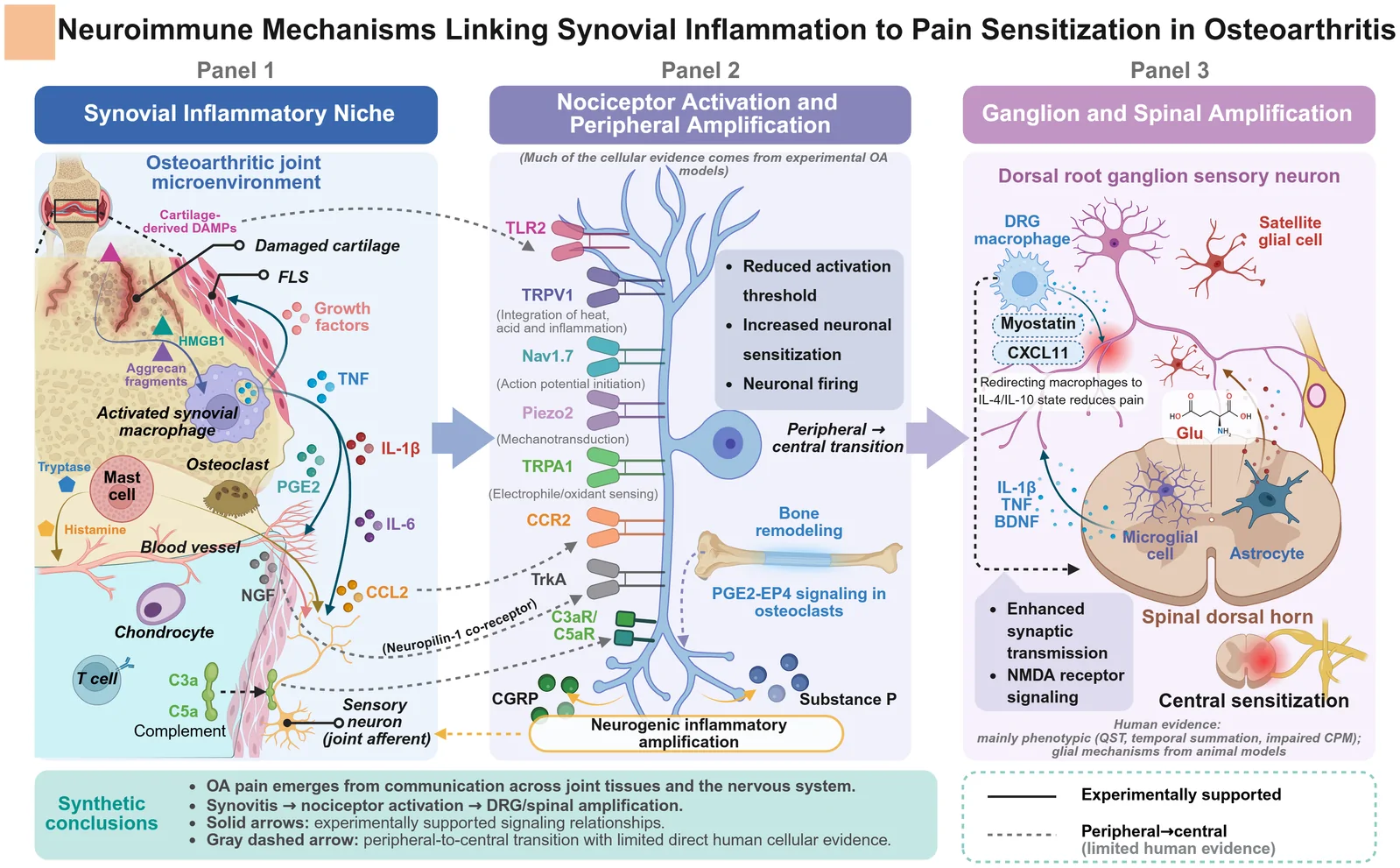
Neuroimmune mechanisms linking synovial inflammation to pain sensitization in osteoarthritis. Macrophages, FLS, mast cells, T cells, and cartilage-derived danger signals generate cytokine, chemokine, lipid, complement, and NGF inputs that converge on nociceptor receptors and channels. TRPV1 integrates heat, acidity, and inflammatory/NGF signaling; TRPA1 detects reactive electrophiles and oxidative stress; Piezo2 mediates mechanical transduction; and Nav channels support action-potential firing. Substance P and CGRP provide feedback to the synovial and vascular niche. Persistent input may recruit DRG macrophages and spinal glia. Solid arrows denote established or experimentally supported signaling relationships; the gray dashed arrow denotes progression from peripheral input to central sensitization, for which human cellular evidence remains limited. BDNF, brain-derived neurotrophic factor; CCL2, C-C motif chemokine ligand 2; CCR2, C-C chemokine receptor 2; CGRP, calcitonin gene-related peptide; DAMP, damage-associated molecular pattern; DRG, dorsal root ganglion; FLS, fibroblast-like synoviocyte; HMGB1, high-mobility group box 1; IL, interleukin; Nav, voltage-gated sodium channel; NGF, nerve growth factor; PGE2, prostaglandin E2; TLR2, toll-like receptor 2; TNF, tumor necrosis factor; TrkA, tropomyosin receptor kinase A; TRPA1, transient receptor potential ankyrin 1; TRPV1, transient receptor potential vanilloid 1.

Sensitized afferents return signals to the joint. Substance P and calcitonin gene-related peptide (CGRP) alter vascular tone, permeability, immune-cell behavior, and matrix responses, creating a neurogenic amplification loop (32). Neuropeptide-positive fibers are present near inflamed human OA synovium, although histological proximity alone does not prove that neurogenic inflammation is the dominant pain mechanism (33). PGE2-EP4 signaling in subchondral osteoclasts and complement-derived C3a/C5a further connect inflammation, bone remodeling, and sensory activation (24, 34). **Figure 1** integrates these convergent and feedback pathways.

## 4 Peripheral neuroimmune amplification and joint tissue crosstalk

### 4.1 Cartilage and subchondral bone

Cartilage and subchondral bone provide distinct but interacting sources of peripheral input. Cartilage-derived DAMPs can activate synovial cells and sensory neurons (23), while macrophage- and FLS-derived mediators accelerate matrix loss and sensitize afferents (8, 9). In subchondral bone, microdamage, pressure change, vascular remodeling, and biochemical signals accompany altered turnover. Osteoclast-derived netrin-1 promotes sensory innervation and pain behavior in experimental OA, linking bone remodeling to nerve growth (35). Human PET-MRI shows spatial adjacency between metabolically active bone and synovitis, but cross-sectional imaging cannot establish which process comes first (36).

### 4.2 Adipose, fibrotic, and neurovascular amplification

The infrapatellar fat pad and systemic metabolic environment add a second layer of regulation. Adipocytes, macrophages, vascular cells, and fibroblasts release adipokines, lipids, cytokines, and complement components. Obesity can therefore intensify OA pain through both load-dependent and metabolic mechanisms, with effects that vary by joint site and sex (37). Adipose-derived leptin and complement factor D provide mechanistic links between adipose dysfunction, disease severity, and pain, but neither pathway alone explains the obesity-associated phenotype (38).

### 4.3 Mechanical-inflammatory feedback

Fibrosis and angiogenesis can stabilize the peripheral amplification loop. Fibrotic FLS subsets alter matrix stiffness and may create permissive routes for vessel and sensory-nerve growth; CD34-high fibroblast subsets are associated with fibrotic features in human knee OA synovium (39). Neovascularization facilitates immune-cell access and often accompanies sensory ingrowth, while hypoxia and mechanical stress reshape stromal mediator production (31, 40). These changes may be especially important around osteophytes, subchondral lesions, and inflamed synovial folds.

Mechanical loading repeatedly perturbs this niche. Compression and shear release matrix fragments and induce prostaglandins or chemokines; once afferents are sensitized, the same movement generates a larger neural response. Mechanical and inflammatory pain are therefore interacting dimensions rather than mutually exclusive categories. Substance P and CGRP further alter blood flow, endothelial permeability, immune-cell behavior, and matrix responses (32). An overactive neuroimmune transcriptional signature in human OA synovium supports the existence of this circuit, although it does not by itself establish direction or causality (41). The most defensible model is a feedback system in which structural damage, immune activation, and neuronal sensitization remain partly independent but mutually reinforcing.

## 5 From peripheral input to central sensitization

### 5.1 DRG and spinal neuroimmune mechanisms

Sustained joint input can recruit pain-maintaining mechanisms beyond the joint. In the DRG, sensory neuron cell bodies interact with resident macrophages and satellite glial cells. Experimental OA studies show that a DRG macrophage program can maintain pain after overt joint inflammation has subsided and that redirecting these cells toward an IL-4/IL-10-responsive state reduces persistent pain behavior (42). Myostatin and CXCL11 also support pain-promoting macrophage states in neural tissue (43). These findings extend the neuroimmune circuit anatomically from joint to ganglion, but equivalent longitudinal cell-state data are not yet available from human DRG.

In the spinal dorsal horn, repeated afferent firing enhances excitatory transmission, weakens inhibitory control, and promotes NMDA-receptor signaling. Activated microglia can release IL-1β, TNF, and brain-derived neurotrophic factor (BDNF), altering neuronal chloride homeostasis and synaptic gain; astrocytes may sustain a later and more persistent state. Microglial activation has been demonstrated in a murine surgical OA model (44), and both microglia and astrocytes contribute to chronic pain behavior in the monosodium iodoacetate model (45). These mechanisms should not be generalized uncritically because chemical injury, surgical instability, and spontaneous OA generate different immune and neural responses.

### 5.2 Human sensitization phenotypes and reversibility

Human evidence is stronger for the clinical phenotype of sensitization than for any specific glial mechanism. Neuropathic pain-like features before knee arthroplasty are associated with altered brainstem pain regulation and a higher risk of persistent postoperative pain (46). In severe hip OA, cerebrospinal-fluid monoamine concentrations correlate with pain and sensitization measures (47). Quantitative sensory testing can detect local hyperalgesia, temporal summation, and impaired conditioned pain modulation, but distributions overlap across patients and are influenced by sleep, mood, and comorbidity (48). A small multimodal study linked intra-articular inflammatory and neurotrophic features with peripheral and central sensitization measures, but its sample size precludes definitive biomarker claims (49).

Central amplification is dynamic rather than inevitably irreversible. Some pain-processing measures normalize after arthroplasty or effective conservative treatment reduces peripheral input, whereas widespread pain can persist despite adequate correction of joint pathology. Longitudinal studies are therefore needed to distinguish reversible gain from established nociplasticity and to identify the stage at which joint-directed immune therapy can still alter central processing. This timing may help explain why the same pathway appears actionable early in disease but ineffective after persistent neural plasticity has developed.

## 6 Therapeutic implications and emerging targets

### 6.1 Target validation, heterogeneity, and safety

Therapy should be matched to the dominant pain mechanism rather than assuming that one anti-inflammatory strategy applies to all patients. NGF blockade provides the clearest clinical validation of a peripheral sensitization target: subcutaneous tanezumab improved pain and function in phase III hip and knee OA studies (50). This benefit was offset by rapidly progressive OA and other joint safety events, with greater risk at higher exposure and in long-term comparisons with nonsteroidal anti-inflammatory drugs (51). The NGF experience therefore establishes both target validity and a narrow safety window. Partial modulation of TrkA-associated signaling, local NGF production, or co-receptors such as neuropilin-1 may be more selective, but protective nociception and joint homeostasis must be preserved.

### 6.2 Why anti-inflammatory trials diverge

Broad immunosuppression has produced inconsistent results, as expected in a disease with low-grade and heterogeneous inflammation. In MRI-selected hand OA with synovitis, methotrexate produced a moderate and potentially clinically meaningful reduction in pain at 6 months (52). In contrast, a 215-participant trial in inflammatory knee OA with effusion found no improvement in pain or synovitis over 52 weeks (53). The different joint sites, entry criteria, doses, and outcome structures make these trials evidence for phenotype dependence, not simply contradictory verdicts on the drug. Local adeno-associated virus-mediated IL-1 receptor antagonist delivery achieved sustained synovial-fluid expression with acceptable phase I safety in nine participants, but controlled efficacy data are still required (54).

### 6.3 Emerging targets and translational maturity

Macrophages, mast cells, chemokines, complement, and glial pathways remain predominantly preclinical targets, and indiscriminate cell depletion may impair repair or inflammation resolution. Neural targets include TRPV1/TRPA1, Piezo2, Nav channels, neuropeptide signaling, and peripheral neurovascular remodeling. Local delivery may reduce systemic exposure, and mechanism-based combinations may interrupt more than one arm of the feedback loop. Nevertheless, pain and structural outcomes should be assessed separately because they need not change synchronously (55). Inhibiting nerve or vessel growth also requires caution because both processes contribute to tissue maintenance and repair (40). Current pharmacotherapy therefore remains mainly symptomatic, and evidence for most neuroimmune combinations is preliminary (56).

### 6.4 Mechanism-informed stratification and trial design

No single biomarker can resolve the major contributors to OA pain. An inflammation-dominant profile may combine contrast-enhanced MRI or ultrasound evidence of synovitis/effusion with soluble markers. Local pressure thresholds and movement-evoked pain can index peripheral sensitization, whereas widespread sensitivity, temporal summation, impaired conditioned pain modulation, sleep disturbance, and neuropathic pain-like descriptors may indicate a nociplastic contribution (57). Bone marrow lesions, instability, and other structural pain sources should be assessed in parallel. Trials should integrate imaging, soluble biomarkers, quantitative sensory testing, and repeated clinical outcomes, with prespecified inflammatory, structural, peripheral-sensitization, and nociplastic subgroups. Mechanism selection should complement, not replace, multimodal care (58).

Timing should be prespecified in translational studies. Suppressing a transient inflammatory response soon after joint injury is biologically different from inhibiting the same pathway after peripheral and central plasticity are established. Paired baseline and on-treatment measurements should confirm target engagement in the intended tissue. When pain does not improve despite engagement, the result can then be interpreted as pathway insufficiency or phenotype mismatch rather than undifferentiated drug failure (**Table 1**).

**Table 1:** Key immune and neuroimmune mediators involved in osteoarthritis pain sensitization.

Source/cell type	Representative mediators	Main target	Pain-related effect	Therapeutic implication
Synovial macrophages	TNF, IL-1β, IL-6, CCL2, PGE2	Nociceptors, FLS, immune cells	Peripheral sensitization and inflammatory amplification	Phenotype-aware macrophage or cytokine/chemokine modulation
FLS/synovial fibroblasts	NGF, IL-6, CXCL chemokines, prostaglandins	Sensory nerves and immune cells	Nerve sensitization, leukocyte recruitment, fibrosis	Local NGF or stromal-inflammatory targeting
Mast cells/lymphocytes	Histamine, tryptase, cytokines, neurotrophic signals	Nociceptors, vessels, stromal cells	Episodic amplification and vascular permeability	Biomarker-selected mediator blockade; human causal evidence limited
Damaged cartilage/crystals	Aggrecan fragments, HMGB1, BCP/CPP crystals, complement signals	TLR2, inflammasome, and complement pathways	Sterile inflammation and direct or indirect nociceptor sensitization	DAMP-, crystal-, TLR-, or complement-directed modulation
Subchondral bone/fat pad	Netrin-1, PGE2, adipokines, complement factor D	Sensory nerves, vessels, immune cells	Nerve growth and metabolic-inflammatory amplification	Bone-remodeling or metabolic phenotype-directed treatment
Nociceptor receptors/channels	TrkA, CCR2, TLR2; TRPV1, TRPA1, Piezo2, Nav1.7	Inflammatory, trophic, mechanical, and damage signals	Threshold lowering, action-potential firing, and peripheral sensitization	Context-selective receptor or channel modulation
Nociceptor-derived feedback	Substance P, CGRP, CCL2	Synovium, vessels, and immune cells	Neurogenic inflammation and feedback amplification	Neuropeptide or CCR2 targeting
DRG macrophages/spinal glia	IL-1β, TNF, BDNF, CXCL11 and related signals	Sensory somata and dorsal horn circuits	Persistent and central sensitization	Glial or neuroimmune modulation; currently mainly preclinical

AAV, adeno-associated virus; BCP, basic calcium phosphate; BDNF, brain-derived neurotrophic factor; CCL2, C-C motif chemokine ligand 2; CCR2, C-C chemokine receptor 2; CGRP, calcitonin gene-related peptide; CPP, calcium pyrophosphate; CXCL, C-X-C motif chemokine ligand; DAMP, damage-associated molecular pattern; DRG, dorsal root ganglion; FLS, fibroblast-like synoviocytes; HMGB1, high-mobility group box 1; IL, interleukin; Nav, voltage-gated sodium channel; NGF, nerve growth factor; PGE2, prostaglandin E2; TLR, Toll-like receptor; TNF, tumor necrosis factor; TRP, transient receptor potential.

## 7 Conclusion

OA pain emerges from communication across joint tissues and the nervous system. Synovitis can initiate or amplify nociception through macrophages, FLS, mast cells, cartilage-derived DAMPs, and calcium-containing crystals. Cytokine, chemokine, lipid, complement, and NGF pathways then converge on nociceptor effectors including TRPV1, TRPA1, Piezo2, and Nav1.7. Sensory neurons feed back through neuropeptides, while subchondral bone, adipose tissue, fibrosis, and neurovascular remodeling extend the peripheral circuit. DRG macrophages and spinal glia may help maintain pain, but the cellular basis of central neuroimmune signaling remains supported more strongly by animal models than by human OA studies.

The translational objective is not to relabel all OA pain as inflammatory, but to determine when an active neuroimmune circuit is clinically dominant and when structural, mechanical, or nociplastic mechanisms prevail. Progress will require spatially resolved human tissue studies, longitudinal sampling, functional validation, and trials enriched for prespecified pain phenotypes. Integrating imaging, soluble biomarkers, quantitative sensory testing, and clinical features may enable rational combinations of local immune modulation with neural, structural, and behavioral therapies.
